# Regularized ensemble learning for prediction and risk factors assessment of students at risk in the post-COVID era

**DOI:** 10.1038/s41598-024-66894-1

**Published:** 2024-07-13

**Authors:** Zardad Khan, Amjad Ali, Dost Muhammad Khan, Saeed Aldahmani

**Affiliations:** 1 Department of Statistics and Business Analytics, United Arab Emirates University, Al Ain, UAE; 2 Department of Statistics, Abdul Wali Khan University Mardan, Mardan, Pakistan

**Keywords:** Ensemble learning, Students at risk, Classification, Face-to-face learning, COVID-19, Mathematics and computing, Statistics

## Abstract

The COVID-19 pandemic has had a significant impact on students’ academic performance. The effects of the pandemic have varied among students, but some general trends have emerged. One of the primary challenges for students during the pandemic has been the disruption of their study habits. Students getting used to online learning routines might find it even more challenging to perform well in face to face learning. Therefore, assessing various potential risk factors associated with students low performance and its prediction is important for early intervention. As students’ performance data encompass diverse behaviors, standard machine learning methods find it hard to get useful insights for beneficial practical decision making and early interventions. Therefore, this research explores regularized ensemble learning methods for effectively analyzing students’ performance data and reaching valid conclusions. To this end, three pruning strategies are implemented for the random forest method. These methods are based on out-of-bag sampling, sub-sampling and sub-bagging. The pruning strategies discard trees that are adversely affected by the unusual patterns in the students data forming forests of accurate and diverse trees. The methods are illustrated on an example data collected from university students currently studying on campus in a face-to-face modality, who studied during the COVID-19 pandemic through online learning. The suggested methods outperform all the other methods considered in this paper for predicting students at the risk of academic failure. Moreover, various factors such as class attendance, students interaction, internet connectivity, pre-requisite course(s) during the restrictions, etc., are identified as the most significant features.

## Introduction

The use of data mining in education, coined as Educational Data Mining (EDM), has grown tremendously in recent years^1–5^, especially, with the massive amount of data generated through online teaching and learning^6–10^. Meaningful insights could be obtained by analyzing information related to students and their learning performance to achieve the desired learning goals^4,11,12^. Moreover, since Spring 2020, the COVID-19 Pandemic restrictions caused many world institutions to use distance education as an alternative to face-to-face instruction^13–16^. Due to the Pandemic, educational activities requiring physical interactions were canceled or suspended in parts of the world that were less resourced^17^. The auspicious interest in distance learning that raised long before the pandemic, many world institutions relied on the e-learning platforms available during the restrictions^18–21^. In this scenario, various studies have assessed the positive and negative effects of the Pandemic on students’ academic performance^22–24^. However, among other general trends, a primary challenge for students during the pandemic has been the disruption of their study habits. Although, some studies have reported improvement in the academic performance during the COVID-19 restrictions^22,24–26^, students getting used to online learning routines might find it even more challenging to perform well in face-to-face learning. Therefore, it is important to analyze students’ academic performance in relation to various potential risk factors after the COVID-19 restrictions, and predict students at risk of academic failure for necessary/timely instructional and administrative interventions which, consequently, helps improving students’ academic efficiency and performance and the efficacy of higher education^27,28^.

Machine learning (ML) methods such as decision trees, random forest, *k*-nearest neighbours, random forest, support vector machine, neural networks, etc., have achieved significant momentum for predicting students academic performance^29^. These predictions are mainly carried out in the context of classification and regression problems with classification (Fail/Pass) being the most popular^30^. The ML methods used for various educational data have shown varying performances in that data available from educational institutions encompass different patterns. Therefore, finding a universily acceptable algorithm is difficult^29^. Furthermore, due to the diverse nature of students’ academic behaviors, standard machine learning methods might find it hard to give accurate predictions for the students at risk of academic failure. Therefore, this research considers regularized classification tree ensembles for the prediction and classification of students’ academic performance. The regularized tree forests considered in this paper select the most accurate and diverse trees based on their individual and collective performance to reduce the effect of ill-performing trees in the final ensemble. Moreover, identifying various risk factors that are potentially associated with students’ academic performance using the regularized tree forest might also help educational institutions for timely interventions.

A primary objective of this research is to assess the transitory influence occurring after the COVID-19 restrictions. In this regard, we will analyze data from students who completed at least one semester during and after the restrictions. For this purpose data are collected from students who enrolled for a university degree during the COVID-19 restrictions and continued their studies after the confinements. This will help in assessing the impact of the habits adopted during the pandemic on their performance when there were no restrictions anymore. Therefore, this paper has the following two objectives

1. To accurately predict students at risk of academic failure using appropriate ML algorithms. This objective is achieved by selecting the most accurate and diverse trees for the random forest ensemble and discarding those that are negatively affected by the inconsistent patterns in the students’ data.
2. To identify various potential risk factors associated with academic failure in relation to the COVID-19 restrictions. This is done by collecting and analyzing records on various features that are believed to influence students’ performance in the post-COVID era.

The rest of the manuscript is arranged as follows. “Literature review” Section gives a detailed review of the associated literature on the use of machine learning methods for analyzing students’ performance data. “Methods” Section gives a description of the methods used in this paper. The dataset used in this paper is described in “Data description” Section. “Data analysis” Section gives results and discussion. The paper ends with a conclusion given in “Conclusion” Section.

## Literature review

There has been increasing interest in using machine learning (ML) methods to predict the academic performance of students. Machine learning methods such as decision trees, random forest, *k*-nearest neighbours, random forest, support vector machine, neural networks, etc., are widely used for analyzing students performance data. Detailed review of the various machine learning methods used for predicting students performance are given in^31–33^. The authors in^34^ explored the use of various machine learning approaches for predicting students performance in e-learning. They found that the Naive Bayes algorithm outperform the other methods they considered on two example datasets. Similarly, the author in^35^, while considering the complicated interrelationships between variables and factors, used deep learning methods for predicting students performance. Cruz-Jesus et al.^36^ collected 16 demographic variables such as gender, age, availability of computing facility, access to internet, class attendance, the number of courses enrolled, etc. to predict students’ academic performance. They used machine learning methods, such as, logistic regression, support vector machine, random forest and k-nearest neighbours, and achieved classification accuracy from 50 to 81%.

Fernandes et al.^37^ proposed a Gradient Boosting Machine (GBM) machine learning model based on students’ in-term achievement grades and demographic characteristics to predict students’ academic achievement. Their results demonstrated that students academic successes was highly related to attendance, the previous year’s achievement scores, and demographic features such as school, neighbourhood and age. Furthermore, they argued that their insights could be used to develop new policies for preventing failure. Similarly, by analyzing student data collected during registration and other environmental features, Hoffait and Schyns^38^ identified students at risk of academic failure utilizing data mining methods. They also used their approach to assign ranks to the students according to the level of the associated risk of failure. Rebai et al.^39^ presented a machine learning method to determine the key features associated with schools’ academic performance and identified the relationships among the features. Based on a regression tree model, they concluded that school size, class size, competition, gender proportions and parental pressure were the most significant features. Moreover, size of the school and the proportion of girls in a school were identified as significant features based on a random forest model. Similarly, Ahmad and Shahzadi^40^ used Artificial Neural Network (ANN) to predict students at risk of academic failure using various features related to study habits, learning skills and academic interaction. They achieved an overall accuracy of 85% based on the ANN model. Musso et al.^41^ used a machine learning method for predicting students at risk of academic failure utilizing information on their learning strategies, motivation, social support perception, health, socio-demographics, and other characteristics related to academic performance. The showed that features related to learning strategies and background information were the most significant variables in predicting students’ academic performance.

Waheed et al.^42^ exploited artificial neural networks using students’ records related to various demographics and their navigation activities in a learning management system (LMS), and concluded that the proposed model could effectively predict academic performance. Xu et al.^43^ explored the inter-dependency of university students internet usage patterns and their academic performance using machine learning methods. They suggested that features related to Internet connection were the most significant features in regulating students academic performance. Similarly, Bernacki et al.^44^ used the log record in LMS to predict students’ performance in a course. A more recent review of the work done on predicting students performance using machine learning methods is given in^45^. The authors in^46^ used deep learning models for predicting students academic performance using students activities on various e-learning platforms and their emotional well-being. Their suggested Student Academic Performance Predicting (SAPP) system achieved a classification accuracy of 96%. Other related work on predicting academic performance can be found in^47–52^ and the references cited therein.

Associated with the COVID-19 pandemic, a significant amount of research is available in literature on predicting students performance and identifying students at risk via machine learning methods . Some of the examples can be found in^53–55^.^56^ has given a thorough review of the existing literature on Educational Data Mining for enhancing e-learning environments with focus on the most widely used methods, together with predicting student performance and the implications of Covid-19 pandemic on e-learning. The authors in^57^ have used EDM for improving the learning process and providing better services to students. They analyzed data produced in a blended system before, during and after the COVID-19 pandemic and gave insights on how to update the learning process to aid professors and students.^58^ used data collected through a questionnaire to determine the efficacy of info-graphics for teaching a computer course during the COVID-19 pandemic using several state-of-the-art machine learning methods. They found that these methods can effectively determine best practices to predict students performance.^59^ developed a generalizable hybrid machine learning model to identify attributes that regulate students performance prediction during the COVID-19 and achieved an overall accuracy of 98.6%. They compared their model with several other frequently used machine learning algorithms.^60^ used EDM to describe academic data gathered from a Greek university e-government analytic platform and determine the quality of academic outputs learning progress through the years. They collected attributes on 4765 students including 1661 unique students using records on 20 courses taught during the COVID-19 pandemic and used EDM for their analysis and gave insights on how to enhance the learning process and the quality of educational services.

Although, some studies have reported positive changes in students learning performance amid the COVID-19 pandemic^22,24,26^, it is still largely unknown how these students, who completed part of studies during the restriction, perform in the post COVID era. This study is an attempt to identify potential risk factor affecting students academic performance and predict students at risk of failure.

## Methods

Students performance data are usually imbalanced and standard machine learning methods struggle to model such data. Moreover, students performances are diverse in nature due to the different levels of achievements and difficulties. Therefore, application of novel methods needs to be considered.

Regularized tree forest methods have been found effective in many studies^61,62^. Here we propose using the idea in^63^, called optimal trees ensemble (OTE) to prune the original forest for better results. Tree selection based on out-of-bag/sub-sample performance will result in further improvements^64^. Three pruning strategies are considered in this paper.

### Out-of-bag and independent samples based tree selection

Exploiting the idea in^63^, let the given training data be ${\mathcal {L}}=(\mathbf{{X}},\mathbf{{Y}})=\{(\mathbf{{x}}_1,y_1),(\mathbf{{x}}_2,y_2),\ldots ,(\mathbf{{x}}_n,y_n)\}$. The $\mathbf{{x}_i}$ are observations on *d* input features and $y_i$ are dichotomous values (in 0, 1 form) representing two possible classes. OTE partitions ${\mathcal {L}}=(\mathbf{{X}},\mathbf{{Y}})$ into two non-overlapping parts, $\mathcal {L_B}=(\mathbf{{X_B}},\mathbf{{Y_B}})$ and $\mathcal {L_V}=(\mathbf{{X_V}},\mathbf{{Y_V}})$. OTE takes the following steps to form the final tree ensemble.

1. Classification trees are grown on *K* bootstrap samples from $\mathcal {L_B}=(\mathbf{{X_B}},\mathbf{{Y_B}})$, using the random forest approach.
2. The *K* built trees are in increasing order of classification error on out-of-bag samples and *T* top trees with the highest prediction accuracy are selected.
3. Commencing from the top most ranked tree, the *T* chosen trees are combined one at a time and $\mathcal {L_V}=(\mathbf{{X_V}},\mathbf{{Y_V}})$ is used to assess whether the newly added tree reduces classification error, in which case it is selected. If it does not reduce classification error, it is discarded.
4. The chosen trees are combined to form the final classification trees ensemble to predict unseen data.

Although OTE has gained better classification accuracy than its competitors on several benchmark and contrived datasets^63^, it still suffers in small sample situations. It is due to the fact that keeping a subset of observations for internal validation in addition to the OOB sample results in missing important information that might be useful for growing an efficient tree model. Previous studies have shown that the classification models’ efficiency is highly associated with the amount of useful information available in the given training data^65^. To exploit most of the training data information, this paper consider two additional pruning strategies as given below.

### Out-of-bag assessment

This procedure uses out-of-bag (OOB) instances in individual as well as ensemble/collective assessment of the trees. It has been empirically shown that, while taking bootstrap samples, about one third of the given training data are missed out from the samples^66^, and play no role in model construction. These observations can be exploited to serve the additional purpose of assessing models by using them as test instances. Let $R_t$, $t=1,\ldots ,K$ and $\bar{R_t}$ be the bootstrap and the associated OOB sample, respectively; and where $H(R_t)$ is the classification model built on $R_t$. Also assume that ${\widehat{Er}}_t$ is the classification error rate of $H(R_t)$ on $\bar{R_t}$. This method then takes the following steps to build the final ensemble.

1. A total of *K* classification trees are grown by exploiting the random forest algorithm on $R_t, t=1,\ldots ,K$. The error rate ${\widehat{Er}}_t$ is estimated for each model.
2. The *K* trees are arranged in increasing order of ${\widehat{Er}}_t$, and top *T* trees are chosen. Let $H^{Q_1}(.),\ldots H^{Q_T}(.)$, be the ordered trees, from the top to the lowest ranks.
3. Commencing from $H^{Q_1}(.)$, assess $H^{Q_j}(.), j=2,\ldots , T$ one at a time by utilizing the associated OOB sample as the unseen data. Choose $H^{Q_j}(.)$ if
  1 $$\begin{aligned} {\hat{{{\mathcal {B}}}{{\mathcal {S}}}}}^{\langle j+ \rangle } < {\hat{{{\mathcal {B}}}{{\mathcal {S}}}}}^{\langle j- \rangle }, \end{aligned}$$
  where ${\hat{{{\mathcal {B}}}{{\mathcal {S}}}}}^{\langle j- \rangle }$ is the Brier score of the model without the *j*th tree and ${\hat{{{\mathcal {B}}}{{\mathcal {S}}}}}^{\langle j+ \rangle }$ is the Brier score of the model with the *j*th tree. Brier score is estimated as
  2 $$\begin{aligned} \hat{{{\mathcal {B}}}{{\mathcal {S}}}} = \frac{\sum _{i=1}^{\text {size of test data}}\left( y_i-{\hat{P}}(y_i | \mathbf{{X}})\right) ^2}{\text {size of test data}}, \end{aligned}$$
  $y_i \in \{0,1\}$ is the value of the response and ${\hat{P}}(y | \mathbf{{X}})$ is corresponding probability estimate of the ensemble given $\textbf{X}$.
4. The trees thus selected form the final ensemble.

### Sub-samples based tree selection

This method takes sub-samples selected randomly without replacement from the given training data ${\mathcal {L}}$ for building the trees. The remainder of each sub-sample is used for assessing the corresponding tree. Let ${\mathcal {R}}_t$, $t=1,\ldots ,K$ be the sub-sample of size $n^\prime < n$, where *n* is the number of observations in the entire training data, and $\bar{{\mathcal {R}}_t}$ be the corresponding remainder of the sample of size $n-n^\prime$; $H({\mathcal {R}}_t)$ is the *t*th tree built using ${\mathcal {R}}_t$. Further assume that $\widehat{Er\_sub}_t$ is the classification error rate of $H({\mathcal {R}}_t)$ on $\bar{{\mathcal {R}}_t}$. This method then takes the following steps to grow the final ensemble.

1. A total of *K* classification trees are grown on $R_t, t=1,\ldots ,K$ instead of bootstrap samples. The error rate $\widehat{Er\_sub}_t$ is estimated for each model.
2. The *K* trees are arranged in increasing order of $\widehat{Er\_sub}_t$, and the top *T* trees are chosen. Let $H^{Q_1}(.),\ldots H^{Q_T}(.)$, be the top ranked, second ranked and so on, ordered trees.
3. Commencing from the top most ranked tree, the *T* chosen trees are combined one at a time and the remaining sub-sample is used to assess, using the aforementioned Brier score based criterion, whether the newly added tree reduces classification error, in which case it is selected. If it does not reduce classification error, it is discarded
4. The chosen trees are combined for forming the final classification trees ensemble to predict unseen data.

The size $n^\prime$ should be chosen so as to avoid potentially correlated trees in the final ensemble due to the fact that only $n\atopwithdelims ()n^\prime$ combinations of the given training data are possible to build trees.

The other algorithms considered in this paper are *k*- nearest neighbour (*k*-NN), weighted *k*-NN^67,68^, extended neighbourhood rule *k*-NN ensemble (ExNRule)^69^, random forest (RF)^70^, support vector machine (SVM)^71^, neural networks (NN)^72^ and linear discriminant analysis (LDA)^73^.

### Ethics approval and consent to participate

Ethics approval for this study was diligently sought and obtained in accordance with the Helsinki Declaration of 1964. The approval was provided by the Advanced Study Research Board (ASRB), Abdul Wali Khan University Mardan. All participants provided informed consent before participating in this research, ensuring that the principles of autonomy and respect for individuals were upheld.

### Consent for publication

All authors have willingly given their full consent for the publication of this paper. Their contributions and commitment to the research and the dissemination of its findings reflect their dedication to the academic community.

## Data description

**Figure 1:**
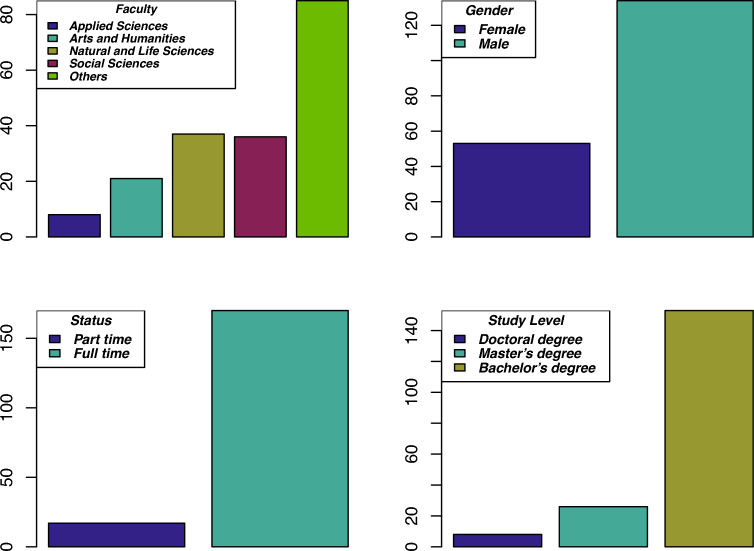
Descriptive representation of $X_1, X_2, X_5, X_6$.

The dataset used in this paper is collected through a comprehensive questionnaire that was administered degree seeking students at, at Abdul Wali Khan University Mardan, Pakistan, who enrolled for their degree program in the years 2020 to 2021 and who had completed part of studies during the pandemic. Information from a diverse group of students is collected encompassing 33 variables covering different aspects of demographic (e.g., age, gender, race, residency status and residency status), socioeconomic and academic profiles. Information on factors related to the COVID-19 restrictions and post-COVID scenario were also collected. The aforementioned variables might influence students academic performance and could be used to predict students at risk of academic failure in the post-COVID era. Table 1 gives the details on all the features considered for this study. Figure 1 display the distribution of study area, gender ratio, status of the students, and the number of students enrolled in various study levels. Students’ GPA in the semester immediately after the COVID restrictions were lifted is used as the response variable. All the other variables are used as predictor variables. A total of 294 responses were collected from the diverse group of students including bachelor, master and PhD students studying in various disciplines. After removing cases with incorrect/missing responses, 245 complete cases are used for the analyses in this paper. All the cases are anonymized due to confidentiality reasons.

The data collected is converted into a binary classification problem by transforming students post-COVID GPA ($X_{28}$) into 0, 1 using a cutoff value of 3.0, i.e.,

$$\begin{aligned} X_{28}= {\left\{ \begin{array}{ll} 1,&{} \text {if } CGPA < 3.0,\\ 0, &{} \text {otherwise.} \end{array}\right. } \end{aligned}$$

This resulted in a binary classification problem with class-wise distribution 44/201, i.e., 44 students with a CGPA in their post-COVID semester(s) less than 3 and 201 students otherwise. This is a highly imbalanced classification problem where the suggested methods are expected to perform better than the rest in that the former discard models that are adversely affected by aforementioned problem. Furthermore, Fig. 1 displays the summary of variables, i.e., faculty of the students ($X_1$), gender ($X_2$), status of the students ($X_5$), and the number of students ($X_6$) enrolled in various study levels.

**Table 1:** List of features and their values.

ID	Variable description	Values
$X_{1}$	Faculty of the student	Arts, applied science, etc.
$X_{2}$	Gender	Male, female
$X_{3}$	Age	Number
$X_{4}$	Nationality	Pakistan, Afghanistan
$X_{5}$	Status	Full or part time
$X_{6}$	Level of study	Bachelor, Master, PhD (Course work only)
$X_{7}$	Academic performance during the COVID-19	Answers from a scale of 5 values
$X_{8}$	Subjects in which student excels academically after COVID-19	Subject name
$X_{9}$	Subjects in which student excels academically during COVID-19	Subject name
$X_{10}$	Completing assignments	Answers from a scale of 5 values (always to never)
$X_{11}$	Reviewing material covered in lecture	Answers from a scale of 5 values (regularly to never)
$X_{12}$	In-class interaction	Answers from a scale of 5 values (always to never)
$X_{13}$	Transition from online to physical learning	Answers from a scale of 4 values (very well to poor)
$X_{14}$	Difficulty in readjusting to the traditional classroom after the COVID-19	Yes, somehow, no
$X_{15}$	Attendance after the COVID-19 restrictions	Regular to irregular
$X_{16}$	Post COVID-19 engagement in learning	Answers from a scale of 4 values
$X_{17}$	Post COVID-19 period collaboration and peers interaction	Positive, negative, no effect
$X_{18}$	Managing workload after the COVID-19	Answers from a scale of 5 values
$X_{19}$	Efficiency in physical exams against online assessments	Improved, no effect, decreased
$X_{20}$	Difficulty concentrating or staying focused in class after the COVID-19	Answers from a scale of 5 values
$X_{21}$	Time management after the COVID-19 restrictions	Answers from a scale of 4 values
$X_{22}$	Effect of face-to-face interaction with teachers and classmates on learning	Positive, negative, no effect
$X_{23}$	Effect of structure daily routine on study	Positive, negative, no effect
$X_{24}$	Access to learning resources after restrictions	Answers from a scale of 5 values
$X_{25}$	Indicator whether a prerequisite courses is taken during the COVID-19	Yes, no
$X_{26}$	Understanding the importance of pre-requisite	Very well, somehow, no
$X_{27}$	Level of difficulty in courses with pre-requisite done during the pandemic	Answers from a scale of 3 values
$X_{28}$	Post COVID GPA	Number
$X_{29}$	GPA in the last semester during COVID-19 restriction	Number
$X_{30}$	Internet connectivity	Answers from a scale of 5 values
$X_{31}$	Class attendance after the COVID-19 restrictions	Number
$X_{32}$	Effect of financial stress due the pandemic on learning	Answers from a scale of 5 values
$X_{33}$	Lack of motivation due to COVID-19 related financial problems	Answers from a scale of 5 values
$X_{34}$	Compromises or sacrifices due to financial constraints	Yes, no
$X_{35}$	Delays or disruptions in studies due to financial difficulty	Yes, no

## Data analysis

### Experimental setup

The experiments in this paper are conducted as follows. The collected data consisted of 245 observations on 33 variables are divided randoly into two non-overlapping parts; a 90% training and a 10% testing parts. The training part of the data is used to build models whereas the testing part is used for assessment purposes. Partitioning into 80% and 70% training, and 20% and 30% testing parts, respectively, is also considered. To stretch the empirical distribution of the desired statistics, the aforementioned random splitting is done 1000 times. Final results reported in the next section, i.e., classification accuracy, Kappa, Sensitivity and Brier score, are the average of all the 1000 runs. All the experiments are done using R programming language.

For the various versions of the suggested optimal trees ensemble (OTE), i.e., OTE-IND, OTE-OOB and OTE-SUB, a total initial ensemble size of 1000 trees is used with the rest of the parameters at their default values as given in the R package OTE^74^. For the random forest ensemble, R package randomForest is used. Its hyper-parameters, i.e., number of trees (ntree), node size (nodesize) and subset size of predictors (mtry) are fine tuned by utilizing tune.randomForest R library as given in the R-Package e1071 using 10-fold cross validation. Support vector machine, the R package kernlab with linear kernel using automatic estimation of the parameter sigma as implemented in the package. *k*-nearest neighbours methods, is fine tuned for the value of the number of nearest neighbours *k*, using tune.knn R function within the R library e1071. All values of $k = 1, \ldots , 10$ are checked to find the best value. For ExNRule method, R package ExNRule is used with the default values of the hyperparameters.

For a fair treatment, the same training and testing data are used for all the methods in all the 1000 runs.

### Results and discussion

Using the above experimental setup, the results from all the method on the dataset are given in Table 2. The table gives the average values of the performance metrics, i.e., classification accuracy, Kappa, sensitivity and Brier score, from the 1000 runs of the various partitions, i.e., 90%/10%, 80%/20% and 70%/30%. Result of the best performing method is shown in bold and the second best method result is given in italic. As can be seen in the table, the suggested regularized tree forest outperformed the other methods in all the cases. OTE-OOB achieved an overall best accuracy of 86.9% in the case of 70% training data, and an accuracy of 87.2% in 90% training case. In the case of 80% training partition, OTE-IND and OTE-OOB gave classification accuracy of 87%. It is evident from the table that classification accuracy increases with increasing size of the training data. Similarly, considering Kappa as performance metric, the regularized optimal trees ensemble gave the best performance achieving 0.701, 0.70 and 0.695 values for 70%, 80% and 90% training partitions, respectively.

In terms of sensitivity, the best performing method is OTE-SUB achieving 73.7%, 76.6% and 73% sensitivities for 70%, 80% and 90% training partitions, respectively. This is due to the fact that, unlike the other two versions of OTE, this method uses most of the training data for building the models as there are only 44 students whose GPA is less than 3. Therefore, in the case of class-imbalanced problem, OTE-SUB is the best method that could be considered for effectively learning patterns from the minority class. Considering Brier score as performance measure, OTE-IND is the best performing method.

In summary, the best and second best performing methods for predicting students at risk of academic failure are the versions of the regularized optimal trees ensemble. In some cases, the results of random forest classifier are close to those of OTE. All the other methods considered in this paper performed poorly.

**Figure 2:**
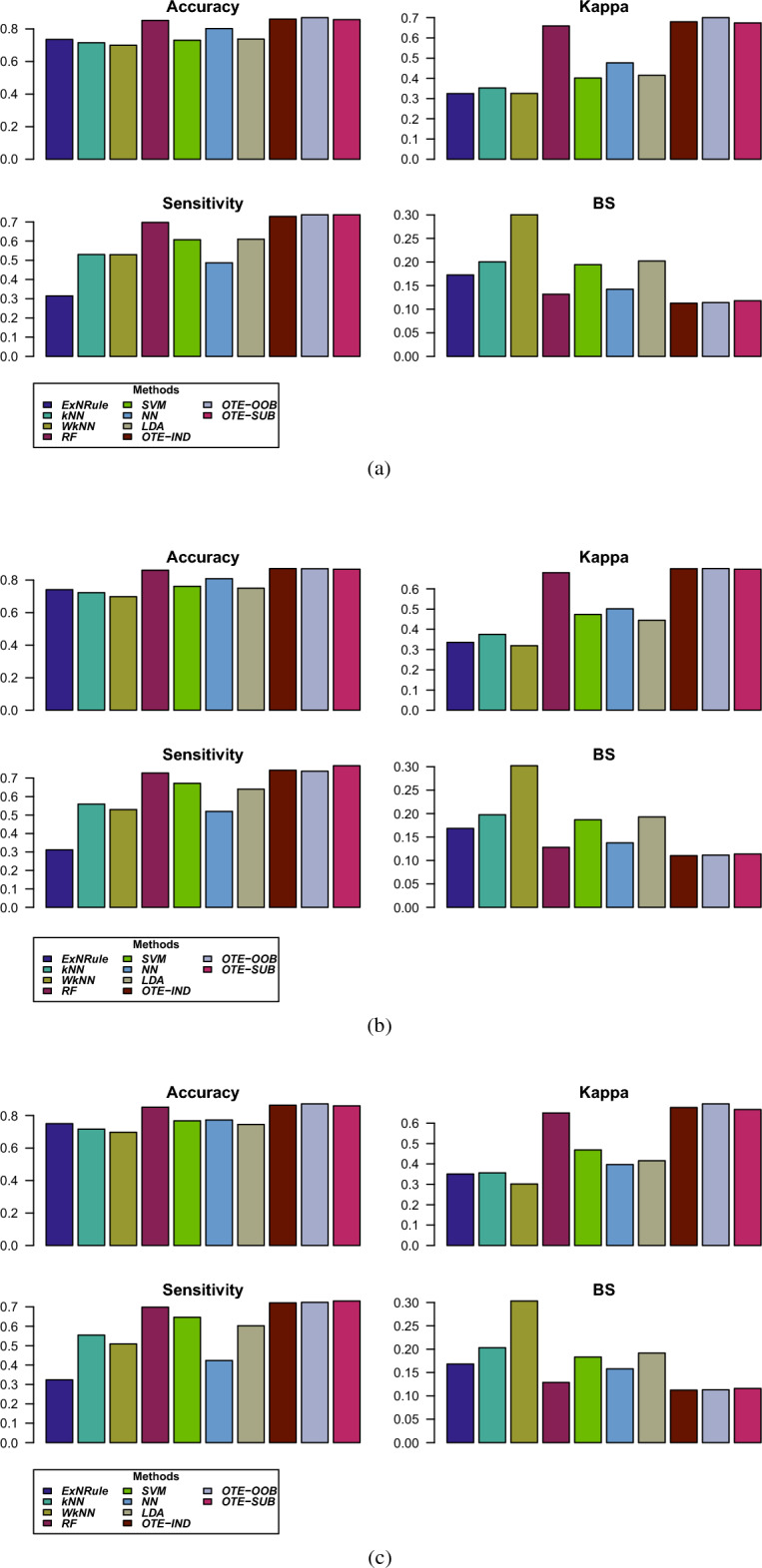
Barplots of the various performance metrics. (**a**) 70% training, (**b**) 80% training, and (**c**) 90% training partition.

**Figure 3:**
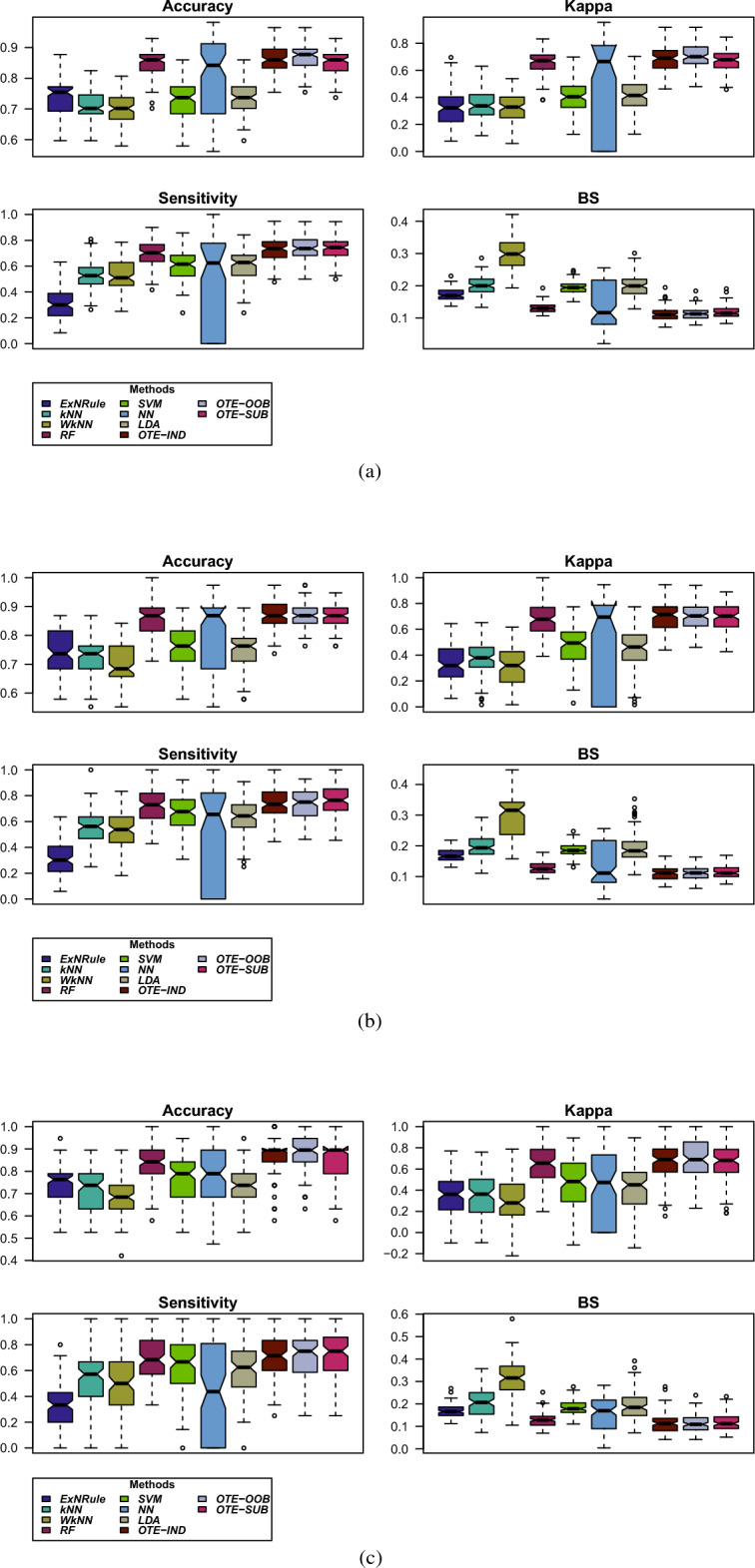
Boxplots of the various performance metrics. (**a**) 70% training, (**b**) 80% training, and (**c**) 90% training partition.

For further illustration, barplots and boxplots of all the performance metrics for the three partitioning schemes are given in Figs. 2 and 3, respectively. Similar conclusion could be drawn from these plots as discussed above.

**Table 2:** Average values of the performance metrics, i.e., classification accuracy, Kappa, Sensitivity and Brier score, from the 1000 runs of the various partitioning, i.e., 90%/10%, 80%/20% and 70%/30% into training/testing parts.

Training data	Metrics	Methods									
		ExNRule	*k*NN	W*k*NN	RF	SVM	NN	LDA	OTE-IND	OTE-OOB	OTE-SUB
$70\%$	Accuracy	0.735	0.715	0.700	0.852	0.730	0.801	0.737	*0.860*	**0.869**	0.856
	Kappa	0.325	0.352	0.325	0.659	0.402	0.477	0.415	*0.679*	**0.701**	0.674
	Sensitivity	0.315	0.530	0.529	0.697	0.607	0.487	0.610	*0.729*	**0.737**	**0.737**
	BS	0.173	0.201	0.300	0.132	0.195	0.142	0.202	**0.113**	*0.114*	0.118
$80\%$	Accuracy	0.741	0.723	0.698	0.861	0.762	0.809	0.750	**0.870**	**0.870**	*0.867*
	Kappa	0.336	0.375	0.319	0.680	0.473	0.501	0.445	**0.700**	**0.700**	*0.697*
	Sensitivity	0.312	0.560	0.530	0.727	0.672	0.520	0.640	0.743	0.737	**0.767**
	BS	0.168	0.198	0.302	0.128	0.187	0.138	0.193	**0.110**	*0.112*	0.114
$90\%$	Accuracy	0.751	0.717	0.697	0.852	0.768	0.773	0.745	*0.864*	**0.872**	0.860
	Kappa	0.351	0.356	0.301	0.650	0.469	0.397	0.416	*0.676*	**0.695**	0.667
	Sensitivity	0.324	0.554	0.509	0.698	0.646	0.424	0.603	0.721	*0.723*	**0.730**
	BS	0.168	0.203	0.303	0.129	0.183	0.158	0.192	**0.112**	*0.113*	0.116

Result of the best performing method is shown in bold and the second best method result is given in italic.

**Figure 4:**
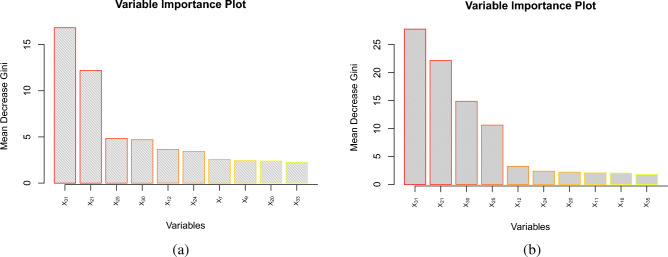
(**a**) Variable importance by random forest. $X_{31}$: Class attendance has the highest importance, $X_{21}$: Time management, $X_{25}$: Taking a pre-requisite course during COVID, $X_{30}$: Internet connectivity, $X_{12}$: In-class interaction, $X_{24}$: Access to learning resources carry high regulatory power. (**b**) Variable importance by OTE. The same set variable identified as random forest with $X_{31}$ and $X_{21}$ causing higher mean decrease in the Gini index than random forest.

For identifying risk factors in predicting students at risk of academic failure, variables importance in terms of the mean decrease in the Gini index caused by each variable is calculated for the random forest and OTE classifiers. Variable importance plots are given in Fig. 4. Figure 4(a) shows variable importance by random forest whereas (b) shows variable importance for the OTE classifier.

It is evident from Fig. 4 that $X_{31}$: class attendance has the highest importance. This feature has also been identified by various other studies as presented in the literature review given in this paper. Similarly, $X_{21}$: time management, $X_{25}$: taking a pre-requisite course during COVID, $X_{30}$: internet connectivity, $X_{12}$: in-class interaction, $X_{24}$: access to learning resources carry high regulatory power. It is interesting to note that taking a pre-requisite course during the COVID-19 restrictions has high regulatory ability due to the change in the mode of learning from online to face-to-face mode. Moreover, internet connectivity might not have an apparent role in predicting students failure in face-to-face learning, however, as the students have completed part of their studies during the restrictions where internet was the basic requirement for effective learning, poor connection to the internet might have caused the formation of a weak foundation in the learning process. The same set of variables are identified by OTE as that of random forest with $X_{31}$ and $X_{21}$ causing more decreasing in the Gini index than random forest. This is due to fact that OTE selects the most accurate and diverse trees, therefore, only important variables cause the highest amount of mean decrease in the Gini index.

## Conclusion

This paper has explored regularized ensemble learning methods for effectively analyzing students performance data and reaching valid conclusions. Three pruning strategies are used for the random forest method that are based on out-of-bag sampling, sub-sampling and sub-bagging. The pruning strategies have been found effective in that trees in the forest that are adversely affected by the unsual patterns in the students data are discarded and only accurate and diverse trees are allowed to form the final ensemble. The regularized methods are applied, in comparison with other state-of-the-art method, such as, *k*- nearest neighbour (*k*-NN), weighted *k*-NN, extended neighbourhood rule *k*-NN ensemble (ExNRule), random forest (RF), support vector machine (SVM), neural networks (NN) and linear discriminant analysis (LDA), on a data collected from university students enrolled for a university degree who have completed part of their studies during the COVID-19 pandemic. The suggested methods outperformed all the other methods considered in this paper for predicting students at risk of academic failure achieving an overall classification accuracy of 87%, Kappa value 0.70, sensitivity 73% and Brier score 0.11. Moreover, various factors such as class attendance, students interaction, internet connectivity, studying a pre-requisite course during the restrictions, etc., are identified as the most regulatory features.

## Data Availability

The datasets used and/or analysed during the current study available from the corresponding author on reasonable request.
